# Prevalence and prognostic value of elevated troponins in patients hospitalised for coronavirus disease 2019: a systematic review and meta-analysis

**DOI:** 10.1186/s40560-020-00508-6

**Published:** 2020-11-23

**Authors:** Bing-Cheng Zhao, Wei-Feng Liu, Shao-Hui Lei, Bo-Wei Zhou, Xiao Yang, Tong-Yi Huang, Qi-Wen Deng, Miao Xu, Cai Li, Ke-Xuan Liu

**Affiliations:** 1 Department of Anaesthesiology, Nanfang Hospital, Southern Medical University, 1838 Guangzhou Ave N, Guangzhou, 510515 China; 2 Department of Medical Ultrasound, Institute of Diagnostic and Interventional Ultrasound, The First Affiliated Hospital, Sun Yat-Sen University, 58 Zhongshan 2nd Road, Guangzhou, 510080 China; 3 Department of Anaesthesiology, The First Affiliated Hospital, Sun Yat-Sen University, 58 Zhongshan 2nd Road, Guangzhou, 510080 China

**Keywords:** Covid-19, Meta-analysis, Myocardial injury, Risk prediction, Troponin

## Abstract

**Background:**

The clinical significance of cardiac troponin measurement in patients hospitalised for coronavirus disease 2019 (covid-19) is uncertain. We investigated the prevalence of elevated troponins in these patients and its prognostic value for predicting mortality.

**Methods:**

Studies were identified by searching electronic databases and preprint servers. We included studies of hospitalised covid-19 patients that reported the frequency of troponin elevations above the upper reference limit and/or the association between troponins and mortality. Meta-analyses were performed using random-effects models.

**Results:**

Fifty-one studies were included. Elevated troponins were found in 20.8% (95% confidence interval [CI] 16.8–25.0 %) of patients who received troponin test on hospital admission. Elevated troponins on admission were associated with a higher risk of subsequent death (risk ratio 2.68, 95% CI 2.08–3.46) after adjusting for confounders in multivariable analysis. The pooled sensitivity of elevated admission troponins for predicting death was 0.60 (95% CI 0.54–0.65), and the specificity was 0.83 (0.77–0.88). The post-test probability of death was about 42% for patients with elevated admission troponins and was about 9% for those with non-elevated troponins on admission. There was significant heterogeneity in the analyses, and many included studies were at risk of bias due to the lack of systematic troponin measurement and inadequate follow-up.

**Conclusion:**

Elevated troponins were relatively common in patients hospitalised for covid-19. Troponin measurement on admission might help in risk stratification, especially in identifying patients at high risk of death when troponin levels are elevated. High-quality prospective studies are needed to validate these findings.

**Systematic review registration:**

PROSPERO CRD42020176747

**Supplementary Information:**

The online version contains supplementary material available at 10.1186/s40560-020-00508-6.

## Introduction

Coronavirus disease 2019 (covid-19) caused by severe acute respiratory syndrome coronavirus-2 (SARS-CoV2) remains a pandemic, with considerable mortality and morbidity exerting pressure on global health-care systems. Patients with covid-19 experience a wide range of disease severity. Prognostic tools that efficiently stratify individual’s risk of experiencing adverse outcomes may facilitate patients and clinicians in the informed decision-making process [1].

Despite being primarily a respiratory infection, covid-19 has important impacts on many vital organs, including the heart [2, 3]. A growing number of reports have documented myocardial injury reflected by elevated circulating cardiac troponin concentrations among infected patients [4–8]. However, elevated troponins frequently occur in patients with conditions other than acute coronary syndromes, and the mechanisms are complex. For this reason, the American College of Cardiology recommended that troponin is ordered for covid-19 patients only when the diagnosis of acute myocardial infraction is being considered on clinical grounds [9]. On the other hand, the UK National Institute for Health and Care Excellence supported troponin testing for wider indications, including patients with non-specific symptoms of possible myocardial injury such as shortness of breath and severe fatigue [10]. Besides, some opinion papers advocated for systematic troponin testing in all covid-19 patients requiring hospital admission for prognostication purpose [11, 12]. These conflicting recommendations regarding the use of troponins in evaluating covid-19 patients reflect major gaps in our understanding of the clinical significance of elevated troponins in this context.

Several recent studies have reported on the relevance of elevated troponins to severity of covid-19 and risk of death, including large retrospective studies from major epicentres such as Wuhan, New York City and some European countries as well as studies with prospective designs [13–16]. We undertook a systematic review and meta-analysis to evaluate the prevalence of elevated troponins in hospitalised covid-19 patients, the performance of elevated troponins in predicting mortality and the quality of currently available evidence.

## Methods

This study was conducted following the modified CHARMS (CHecklist for critical Appraisal and data extraction for systematic Reviews of prediction Modelling Studies) for reviews of prognostic factors (CHARMS-PF) guidelines [17] and reported in accordance with the Preferred Reporting Items for Systematic Reviews and Meta-Analyses (PRISMA) statement (checklist in Table S1) [18]. The study protocol was registered prospectively in PROSPERO (CRD42020176747).

### Literature search and eligibility criteria

We searched English and Chinese databases (PubMed, Embase, Chinese National Knowledge Infrastructure and Chinese Biomedical Database) and preprint servers (medRxiv, bioRxiv, ChinaXiv, Research Square and SSRN) for research articles on covid-19 published after 1 December 2019, with no restrictions on language or peer review status. Details of the search strategy are listed in Table S2. The last update of literature search was performed on 15 October 2020. Studies were considered eligible if they were observational or interventional studies that (1) enrolled patients that required hospitalisation for covid-19 pneumonia, (2) measured cardiac-specific troponin T or I concentrations on hospital admission or during hospital stay and (3) reported the prevalence of elevated troponins among patients who received troponin measurement or the association between troponin concentrations and mortality during the follow-up. Elevated troponins were defined as troponin concentrations higher than the upper reference limit value predetermined by the local laboratory. We excluded (1) studies that specifically enrolled patients admitted for cardiovascular reasons, organ transplant recipients or deceased patients; (2) studies that reported myocardial injury but the diagnosis was not based solely on troponin measurements or the upper reference limit of troponin test was not used as the diagnostic criteria; and (3) case reports or case series involving fewer than 10 patients. When more than one study from the same institutions with overlapping time period of recruitment were identified, we chose the one with the largest sample size for inclusion, except when our outcomes of interest were reported only in smaller studies.

### Study selection, data extraction and quality assessment

Two researchers (BCZ and WFL) independently screened the identified records, first based on the title and abstract and subsequently based on the full manuscript. The reference lists of relevant manuscripts were searched to identify additional eligible studies. Using predefined forms, both researchers independently extracted data on study design, time of recruitment and follow-up, characteristics of patients and troponin tests, frequency of troponin elevation and, if available, the number of survivors and nonsurvivors among patients with and without elevated troponins. For studies that performed multivariable analysis to assess the association between elevated troponins and mortality, we extracted the adjusted effect estimates with confidence intervals (CI) and the variables in multivariable models. Risk of bias assessment for studies reporting on the prevalence of elevated troponins was conducted using a tool developed for prevalence studies [19]. Key study features assessed were sample selection and measurement quality. The Quality in Prognosis Studies (QUIPS) tool was used to assess the risk of bias of studies on the prognostic performance of troponins [20]. The tool includes the evaluation of 6 domains: study participation, study attrition, prognostic factor measurement, outcome assessment, study confounding, and statistical analysis and reporting. Disagreements between researchers were resolved by consensus, and a third researcher was involved when necessary.

### Statistical analysis

Statistical analyses were performed using Stata® version 12.0 (StataCorp, College Station, TX, USA). All meta-analyses were based on random-effects models due to the anticipated high degree of heterogeneity among studies. To estimate the prevalence of elevated troponins in patients admitted for covid-19, we pooled the proportion of patients with elevated troponins among those who received at least one troponin measurement. The Freeman-Tukey double arcsine transformation method was used to account for studies reporting very low prevalence estimates.

To assess the predictive value of troponins for mortality, we pooled the multivariable-adjusted associations between elevated troponins and mortality. For this analysis, studies that did not measure troponins on hospital admission were not included, because troponins are expected to rise in late deterioration of the illness and have less predictive utility at that time. We also excluded studies that modelled troponin as a continuous predictor or did not use the upper reference limit of the troponin test, because the association between troponin concentrations and mortality risk may not be linear and because the use of study-specific optimal cut-off thresholds in meta-analysis may result in overestimation of the prognostic value of a biomarker [21]. We converted adjusted odds ratios reported by included studies to risk ratios (RR) using a published formula [22] and assumed that hazard ratios reasonably approximated RRs. For studies where more than one effect estimates were given for different levels of troponin elevation versus reference, a study-unique effect estimate was generated using a fixed-effects model before being included in the random-effects meta-analysis. We calculated the summary RR with 95% CI using the generic inverse variance method. Considering that dozens of commercial troponin assays with different epitope targets and analytical characteristics are used in clinical practice, we calculated 95% prediction intervals (PI) to inform the distribution of prognostic effects of elevated troponins across different measurement methods in future studies [23].

We evaluated publication bias using the funnel plot and Egger’s test. When significant publication bias was found, we used trim-and-fill method to adjust our results. The magnitude of heterogeneity was assessed by the Higgins *I*^2^ statistic. Subgroup analyses in case of substantial heterogeneity (*I*^2^ > 50%) were conducted based on the characteristics of troponin assays (troponin T or I, high-sensitivity or contemporary assays) and the geographical location, sample size, risk of bias and peer review status of included studies.

The predictive ability of troponin was further assessed by constructing a hierarchical summary receiver operating characteristic curve using the bivariate model [24]. Based on the model, we determined the overall sensitivity, specificity and positive and negative likelihood ratios of elevated troponins on hospital admission for predicting death. These measures of predictive accuracy reflect the intrinsic performance of troponins and are independent of the mortality of underlying study populations. For practical purposes, we estimated the post-test risk of death using the Fagan nomogram, considering a pre-test probability as the pooled risk of death among the included patients.

## Results

### Characteristics of selected studies

**Fig. 1:**
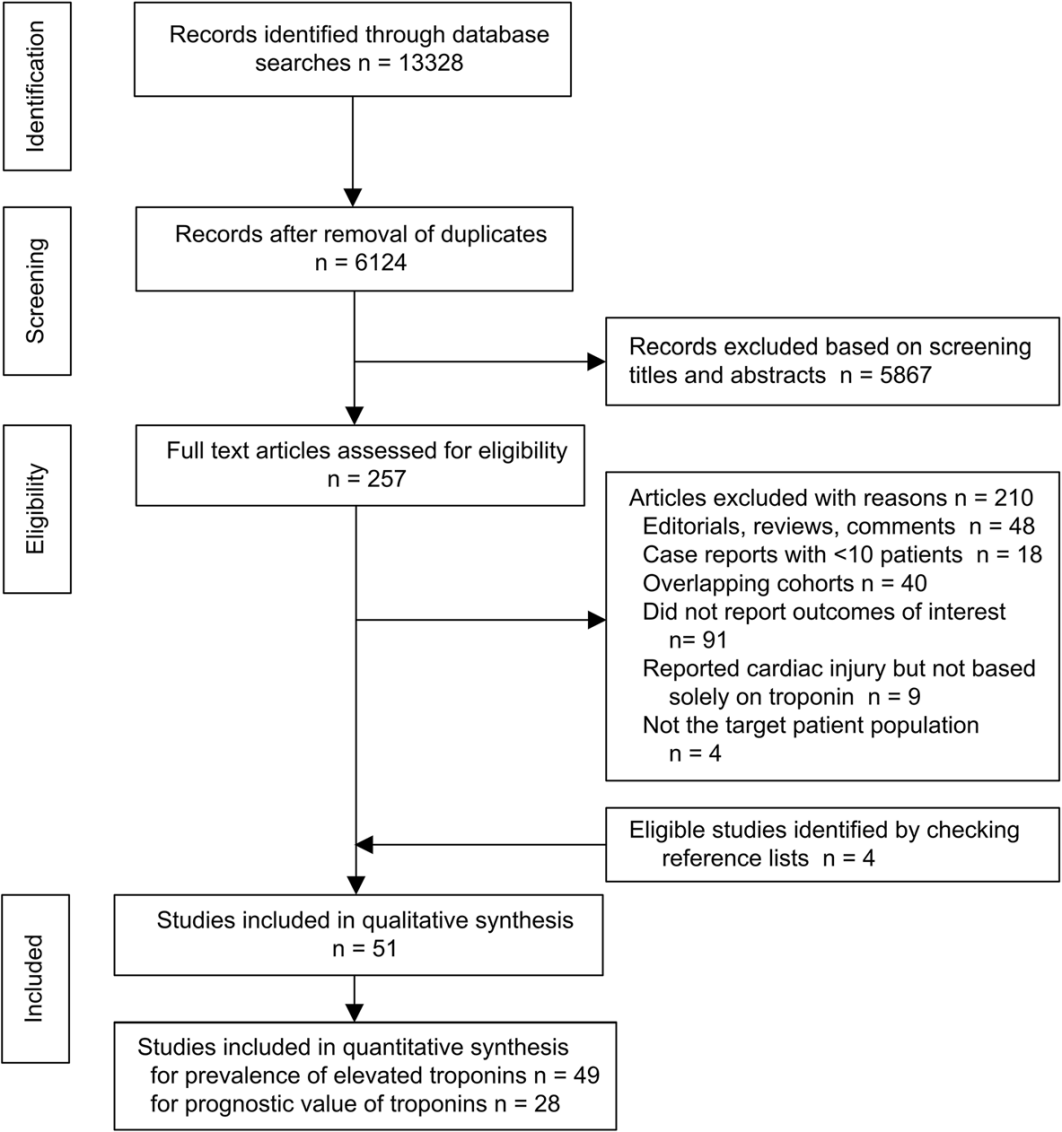
PRISMA flow chart showing study selection process

Of the 6124 unique records identified, 51 studies were selected for this review (Fig. 1, Table 1), including 41 studies that have been peer-reviewed [13–16, 25–61] and 10 published only as a preprint [62–71]. Five were prospective studies [16, 32, 38, 47, 58] and the others were retrospective in design. Patients included in these studies (sample size range, 15–6247; median age, 44–72 years; proportion of men, 43–79%) were admitted to hospitals up to 21 June 2020. Information on methods and findings of troponin measurement in each study were summarised in Table 2.

**Table 1:** Main characteristics of included studies

Study author	Location	Design	Date of admission	Patients		Comorbidities (HTN/DM/CVD, %)	Disease severity^**a**^	Date of last follow-up	Overall mortality, %	Mortality of pts with elevated troponin on admission, %
				No. (male, %)	Age, years					
Arcari et al.	Rome, Italy	Retrospective, multi-centre	Mar 15–Apr 30	111 (45.9)	72 (17)	55.9/18.9/31.5	NA	May 31	20.7	30.8
Azoulay et al.	Paris, France	Retrospective, multi-centre	Feb 21–Apr 24	376 (76.9)	62 [53–68]	49.5/30.3/NA	100% critical, SOFA score 5 [3, 8]	May 15	26.4	NA
Barman et al.	Istanbul, Turkey	Retrospective, multi-centre	Mar 20–Apr 20	607 (55.0)	63 (14)	43.8/31.6/19.1	32.1% critical	Apr 20	17.0	42.7
Bhatla et al.	Philadelphia, USA	Retrospective, single-centre	Mar 6–May 19	700 (44.9)	50 (18)	49.6/26.0/1.6	11.3% critical	May 24	4.3	NA
Bhatraju et al.	Seattle, USA	Retrospective, multi-centre	Feb 24–Mar 9	24 (62.5)	64 (18)	NA/58.3/NA	100% critical	Mar 23	50.0	NA
Buckner et al.	Seattle, USA	Retrospective, multi-centre	Mar 2–Mar 26	105 (50.5)	69 (range 23–97)	59.0/33.3/38.1	48.6% critical	May 8	33.3	NA
Cipriani et al.	Padova, Italy	Retrospective, single-centre	Feb 26–Mar 31	109 (67.0)	70 [60–81]	62.3/24.8/16.5	28.4% critical	Apr 1	18.3	43.9
Du et al.	Wuhan, China	Prospective, single-centre	Dec 25–Feb 7	179 (54.2)	58 (14)	32.4/18.4/16.2	NA	Mar 24	11.7	31.7
Ferguson et al.	North California, USA	Retrospective, multi-centre	Mar 13–Apr 11	72	NA	36.1/27.8/NA	29.2% critical	May 2	6.9	NA
Franks et al.	St. Louis, USA	Retrospective, single-centre	NA	182 (56.6)	64 (range 19–98)	NA	NA	NA	18.7	36.9
Gottlieb et al.	Chicago, USA	Retrospective, single-centre	Mar 4–Jun 21	1483 (53.4)	56 [44–68]	60.5/42.8/15.4	35.6 % critical	Jul 10	10.0	NA
Goyal et al.	New York, USA	Retrospective, multi-centre	Mar 3–Mar 27	393 (60.6)	62 [49, 74]	50.1/25.2/13.7	NA	Apr 10	10.2	NA
Harmouch et al.	Bethlehem, USA	Retrospective, single-centre	Mar 1–Apr 15	563 (57.1)	63	50.3/35.2/NA	24.3 % critical	NA	14.5	36.1
He et al.	Wuhan, China	Retrospective, single-centre	Feb 8–Mar 16	94 (57.4)	69 (10)	59.6/19.1/12.8	100% critical	Mar 16	44.7	NA
Heberto et al.	Puebla and Mexico City, Mexico	Prospective, multi-centre	Mar-Apr	254 (65.7)	54 (13)	35.4/31.5/5.5	NA	To death or discharge	35.0	NA
Hu et al.	Wuhan, China	Retrospective, single-centre	Jan 8–Feb 20	323 (51.4)	61 (range 23–91)	32.5/14.6/2.2	45.2% severe, 8.0 critical	Mar 10	10.8	NA
Huang et al.	Jiangsu, China	Retrospective, multi-centre	Jan 24–Apr 20	60 (58.3)	57 (range 26–97)	23.3/16.7/5.0	100% critical, APACHE II score 14 (5)	Apr 20	0	NA
Karbalai et al.	Tehran, Iran	Retrospective, single-centre	Mar-May	386 (61.1)	59 (16)	36.8/34.5/25.1	20.5 % critical	To death or discharge	19.9	NA
Lala et al.	New York, USA	Retrospective, multi-centre	Feb 27–Apr 12	2736 (59.6)	66	38.9/26.3/16.6	CURB-65 score 1.26 (1.10)	Apr 12	27.3	NA
Lazzeri et al.	Florence, Italy	Retrospective, single-centre	Mar 1–Mar 31	28 (78.6)	62 (10)	89.3/39.3/28.6	100% critical	Mar 31	7.1	NA
Li et al.	Guangzhou, China	Retrospective, single-centre	Jan 24–Feb 25	82 (58.5)	45 (16)	26.8/21.9/NA	29.3% severe or critical	Feb 29	1.2	7.7
Li et al.	Wuhan, China	Retrospective, single-centre	Jan 29–Apr 1	2068 (48.6)	63 [51, 70]	34.9/14.1/8.8	32.0 % critical	Apr 1	8.8	45.9
Lombardi et al.	Italy	Retrospective, multi-centre	Mar 1–Apr 9	614 (70.8)	67 (13)	50.5/19.8/15.0	SOFA score 2 [1, 3]	Apr 23	24.1	37.4
Lorente-Ros et al.	Madrid, Spain	Retrospective, single-centre	Mar 18–Mar 23	707 (62.7)	67 (16)	50.5/20.2/10.6	52.1% critical	1 month after admission	19.8	45.2
Lu et al.	Wuhan, China	Retrospective, single-centre	Dec 30–Mar 18	50 (58.0)	66 [58, 73]	38.0/18.0/18.0	100% critical, APACHE II score 12.0 [9.0, 16.3]	To death or discharge	60.0	NA
Ma et al.	Chongqing, China	Retrospective, single-centre	Jan 21–Mar 2	84 (57.1)	48 [43, 63]	14.3/11.9/6.0	76.2% severe or critical	Mar 2	0	NA
Majure et al.	New York, USA	Retrospective, multi-centre	Mar 1–Apr 27	6247 (59.9)	66 [56, 77]	59.5/36.0/13.3	30.2% critical	Apr 30	22.4	43.5
Mejía-Vilet et al.	Mexico City, Mexico	Prospective, single-centre	Mar 16–May 21	569 (66.3)	D, 49 [41, 60]; V, 52 [43, 60]	28.5/27.8/NA	35.0% critical	May 24	11.1	NA
Nguyen et al.	Chicago, USA	Retrospective, single-centre	Mar 16–Apr 16	356 (48.0)	61 [50, 73]	69.5/42.1/21.9	44% critical	May 25	12.6	21.4
Nie et al.	Wuhan, China	Retrospective, single-centre	Jan 12–Mar 12	311 (61.1)	63 [54, 70]	NA	57.9% severe, 9.6% critical	Mar 20	35.7	NA
Petrilli et al.	New York, USA	Retrospective, multi-centre	Mar 2–Apr 2	1999 (62.6)	62 [50, 74]	37.1/25.2/9.9	36.1% critical	Apr 7	14.6	NA
Price-Haywood et al.	Louisiana, USA	Retrospective, multi-centre	Mar 1–Apr 11	1382 (49.0)	63 (15)	NA	34.3% critical	May 7	23.6	NA
Qi et al.	Chongqing, China	Retrospective, multi-centre	Jan 19–Feb 16	267 (55.8)	48 [35, 65]	7.5/9.7/4.9	18.7% severe or critical	Feb 16	1.5	NA
Qin et al.	Hubei, China	Retrospective, multi-centre	Dec 31–Mar 4	3219 (47.7)	57 [45, 66]	27.8/12.8/6.4	NA	28 days after admission	6.0	37.9
Raad et al.	Michigan, USA	Retrospective, multi-centre	Mar 9–Apr 15	1020 (49.9)	63 [52, 73]	72.7/44.3/12.1	50.2% critical	To death or discharge	17.6	32.8
Shah et al.	Albany, USA	Retrospective, multi-centre	Mar 2–Jun 7	309 (42.7)	63 (14)	84.5/46.3/9.1	35.6% critical	To death or discharge	21.4	NA
Shah et al.	San Francisco, USA	Retrospective, single-centre	Feb 3–Mar 31	26 (NA)	NA	NA	42.3% critical	Apr 25	3.8	NA
Shen et al.	Shanghai, China	Retrospective, single-centre	Jan 20–Feb 29	325 (51.7)	51 [36, 64]	15.6/7.1/3.4	3.1% severe, 4.9% critical	Feb 29	0.9	NA
Stefanini et al.	Milan, Italy	Retrospective, single-centre	Up to Apr 1	397 (67.3)	67 [55, 76]	56.7/24.6/8.4	NA	To death or discharge	23.2	45.4
Szekely et al.	Tel Aviv, Israel	Prospective, single-centre	Mar 21–Apr 16	100 (63.0)	66 (17)	57.0/29.0/16.0	29.0% severe, 10.0% critical	NA	NA	NA
Tan et al.	Wuhan, China	Retrospective, multi-centre	Feb 8–Apr 6	115 (49.6)	63 [55, 70]	47.0/25.2/NA	63.5% severe, 36.5% critical	Apr 6	19.1	70.0
van den Heuvel et al.	Nijmegen, Netherlands	Retrospective, single-centre	Apr 1–May 12	51 (80.4)	63 [51, 68]	41.1/17.6/9.8	37.2% critical	To death or discharge	2.0	NA
Wei et al.	Chengdu, China	Retrospective, multi-centre	Jan 16–Mar 10	101 (53.5)	49 [34, 62]	20.8/13.9/5.0	36.6% severe or critical	NA	3.0	18.8
Woo et al.	Philadelphia, USA	Retrospective, multi-centre	Mar 1–Apr 30	415 (55.2)	65 (15)	69.9/35.0/18.1	39.3% critical	14 days after admission	21.2	NA
Xu et al.	Guangzhou, China	Retrospective, single-centre	Jan 22–Apr 6	15 (60.0)	44 (13)	26.7/NA/NA	53.3% severe, 6.7% critical	Apr 6	0	NA
Yang et al.	Wuhan, China	Retrospective, single-centre	Feb 13–Mar 14	463 (49.9)	60 [50, 69]	38.7/17.3/7.3	8.2% severe, 14.3% critical	Mar 28	5.8	40.0
Yu et al.	Wuhan, China	Prospective, multi-centre	Feb 25–Feb 27	226 (61.5)	64 [57, 70]	42.5/20.8/9.7	100% critical, SOFA score 4 [2, 8]	Apr 9	38.5	NA
Zeng et al.	Shenzhen, China	Retrospective, single-centre	Jan 11–Apr 1	416 (47.6)	NA	14.4/5.5/3.1	8.4% critical	Apr 1	0.7	NA
Zhang et al.	Wuhan, China	Retrospective, single-centre	Feb 1–Mar 15	135 (49.6)	56 [42, 68]	20.7/11.9/3.0	22.2% critical	NA	13.3	35.0
Zhao et al.	Jingzhou, China	Retrospective, single-centre	Jan 16–Feb 10	91 (53.8)	46	19.8/3.3/0	33.0% severe or critical	Feb 10	2.2	NA
Zhou et al.	Wuhan, China	Retrospective, multi-centre	Dec 29–Jan 31	191 (62.3)	56 [46, 67]	30.4/18.8/7.9	34.6% severe, 27.7% critical	Jan 31	28.3	95.8

*APACHE* Acute Physiology and Chronic Health Evaluation; *CVD* Cardiovascular disease; *CURB-65* Confusion, Urea nitrogen, Respiratory rate, Blood pressure, 65 years of age and older; *D* Derivation cohort; *DM* Diabetes mellitus; *HTN* Hypertension; *ICU* Intensive care unit; *NA* Not available; *SOFA* Sequential Organ Failure Assessment; *V* Validation cohort

Values were mean (standard deviation), median [interquartile rage] or median (range)

^a^Severity of illness was assessed based on covid-19 treatment guidelines (see supplementary Table S7)

**Table 2:** Information on troponin measurement in included studies

Study	Type of Tn assay	Manufacturer	Upper reference limit	Time of measurement	Proportion of patients with Tn measurement, no. measured/no. enrolled (%)	Frequency of Tn elevation, no. elevated/no. measured (%)
Arcari et al.	hs-TnT, hs-TnI	Roche, NA	14 ng/L, 35 ng/L	Within 24 h of admission	103/111 (92.8)	39/103 (37.9)
Azoulay et al.	NA	NA	NA	On ICU admission	343/379 (90.5)	135/343 (39.4)
Barman et al.	hs-TnI	NA	14 ng/L	On admission	607/908 (66.9)	150/607 (24.7)
Bhatla et al.	NA	NA	0.010 ng/mL	On admission	373/700 (53.3)	82/373 (22.0)
Bhatraju et al.	NA	NA	0.06 ng/mL	During first 3 days in ICU	13/24 (54.2)	2/13 (15.4)
Buckner et al.	NA	NA	0.1 ng/mL	During hospitalisation	67/105 (63.8)	13/67 (19.4)
Cipriani et al.	hs-TnI	NA	32 ng/L for men, 16 ng/L for women	On admission and during hospitalisation	109/136 (80.1)	On admission, 41/109 (37.6); overall, 46/109 (42.2)
Du et al.	TnI	NA	0.05 ng/mL	On admission	179/179 (100)	41/179 (22.9)
Ferguson et al.	NA	NA	0.055 ng/mL	Within 24 h of admission	45/72 (62.5)	2/45 (4.4)
Franks et al.	TnI	Abbott	0.03 ng/mL	On admission and during hospitalisation	On admission, 128/182 (70.3); overall, 143/182 (78.6)	On admission, 65/128 (50.8); overall, 80/143 (55.9)
Gottlieb et al.	NA	NA	0.9 ng/mL	On admission	390/1483 (26.3)	87/390 (22.3)
Goyal et al.	NA	NA	0.5 ng/mL	Within 48 h of admission	246/393 (62.6)	11/246 (4.5)
Harmouch et al.	TnI	NA	0.05 ng/mL	On admission	482/560 (86.1)	97/482 (20.1)
He et al.	hs-TnI	NA	NA	During stay in ICU	94/94 (100)	35/94 (37.2)
Heberto et al.	hs-TnI	Beckman	17.5 ng/L	On admission	254/254 (100)	64/254 (25.2)
Hu et al.	TnI	Siemens	0.040 ng/mL	On admission	323/323 (100)	68/323 (21.1)
Huang et al.	TnT	NA	0.13 ng/mL	On ICU admission	60/60 (100)	19/60 (31.7)
Karbalai et al.	hs-cTnI	NA	26 ng/L for men, 11 ng/L for women	During hospitalisation	386/386 (100)	115/386 (29.8)
Lala et al.	TnI	Abbott	0.03 ng/mL	Within 24 h of admission	2736/3047 (89.8)	1751/2736 (36.0)
Lazzeri et al.	TnT	NA	0.028 ng/mL	On ICU admission	28/28 (100)	11/28 (39.3)
Li et al.	TnI	Beckman	0.03 ng/mL	On admission	82/82 (100)	13/82 (15.9)
Li et al.	hs-TnI	Abbott	34.2 ng/L	On admission	2068/2699 (76.6)	181/2068 (8.8)
Lombardi et al.	NA	NA	NA	Within 24 hours of admission	614/614 (100)	278/614 (45.3)
Lorente-Ros et al.	hs-TnI	NA	14 ng/L	On admission	707/707 (100)	148/707 (20.9)
Lu et al.	TnI	NA	0.4 ng/mL	During stay in ICU	50/72 (69.4)	36/50 (72.0)
Ma K, et al	TnI	NA	0.034 ng/mL	During hospitalisation	84/84 (100)	9/84 (10.7)
Majure et al.	TnI, TnI, TnT, hs-TnT	Siemens, Siemens, Roche, Roche	0.045 ng/mL, 0.056 ng/mL, 0.01 ng/mL, 19 ng/L	Within 48 hours of admission	6247/11,159 (56.0)	1821/6247 (29.1)
Mejía-Vilet et al.	TnI	NA	0.020 ng/mL	On admission	569/569 (100)	86/569 (15.1)
Nguyen et al.	hs-TnI	NA	22 ng/L	On admission	340/356 (95.5)	140/340 (41.2)
Nie et al.	hs-TnI	Abbott	26.2 ng/L	During hospitalisation	NA	103/311 (33.1)
Petrilli et al.	NA	NA	0.1 ng/mL	On admission	2510/2729 (92.0)	NA
Price-Haywood et al.	TnI	NA	0.06 ng/mL	On admission	1084/1382 (78.4)	270/1084 (24.9)
Qi et al.	hs-TnT	Roche	14 ng/L	On admission	76/267 (28.5)	3/76 (3.9)
Qin et al.	hs-TnI, TnI	Various	Various	On admission	1462/6033 (24.2)	95/1462 (6.5)
Raad et al.	hs-TnI	Beckman	18 ng/L	On admission	1020/1044 (97.7)	390/1020 (38.2)
Shah et al.	TnI	NA	0.05 ng/mL	During hospitalisation	309/635 (48.7)	116/309 (37.5)
Shah et al.	TnI	NA	0.05 ng/mL	During hospitalisation	14/26 (53.8)	5/14 (35.7)
Shen et al.	TnI	Siemens	0.040 ng/mL	On admission	325/325 (100)	80/325 (24.6)
Stefanini et al.	hs-TnI	Beckman	19.6 ng/L	On admission	397/397 (100)	130/397 (32.7)
Szekely et al.	TnI	Abbott	28 ng/L	On admission	100/100 (100)	20/100 (20)
Tan et al.	hs-TnI	Abbott	26.2 ng/L	On admission	115/115 (100)	20/115 (17.4)
van den Heuvel et al.	hs-TnT	Roche	14 ng/L	During hospitalisation	47/51 (92.2)	24/47 (51.1)
Wei et al.	hs-TnT	Roche	14 ng/L	On admission	101/103 (98.1)	16/101 (15.8)
Woo et al.	hs-TnT, TnI	Roche, Siemens	19 ng/L, 0.040 ng/mL	On admission	NA	NA
Xu et al.	TnI	NA	NA	On admission	15/15 (100)	1/15 (6.7)
Yang et al.	TnI	Siemens	0.040 ng/mL	On admission	463/463 (100)	45/463 (9.7)
Yu et al.	hs-TnI, TnI	Abbott, NA	28 ng/L, 0.3 ng/mL	During stay in ICU	226/226 (100)	61/226 (27.0)
Zeng et al.	TnI	NA	0.026 ng/mL	On admission	345/416 (82.9)	29/345 (8.4)
Zhang et al.	hs-TnT	Roche	14 ng/L	On admission	135/135 (100)	40/135 (29.6)
Zhao et al.	TnI	NA	0.01 ng/mL	On admission	88/91 (96.7)	3/88 (3.4)
Zhou et al.	hs-TnI	Abbott	28 ng/L	On admission	145/191 (75.9)	24/145 (16.6)

*Hs* High-sensitivity, *ICU* Intensive care unit, *NA* Not available, *Tn* Troponin

### Prevalence of elevated troponins

In total, 49 studies reported or allowed calculation of prevalence estimates for elevated troponins above the upper reference limit in patients hospitalised for covid-19. The risks of bias of these studies in estimating prevalence are summarised in Table S3. The studies differed in patient population (patients admitted to hospital or patients admitted in ICU) and timing of troponin measurement (on admission or during hospital stay). We decided to conduct separate analyses for studies using different methodologies because these may have significant influence on the observed prevalence of elevated troponins.

In 35 studies (22,473 patients) where the prevalence of elevated troponins at the time of hospital admission could be extracted [13–16, 25, 27, 28, 31–39, 43–47, 50, 51, 54, 55, 57, 59–61, 65–67, 69–71], the pooled estimate was 20.8% (95% CI 16.8–25.0 %) with substantial heterogeneity (*I*^2^ = 98.0%) (Figure S1). No publication bias was suggested by the funnel plot (Figure S2) or the Egger’s test (*P* = 0.292). When we exclude studies that measured troponin in less than 90% of consecutively admitted patients, the remaining 19 studies (5930 patients) deemed at low risk of selection bias yielded a pooled prevalence of 22.9% (95% CI 17.6–28.6 %).

In 9 studies (1470 patients) [30, 31, 34, 41, 48, 52, 53, 56, 64], the prevalence of elevated troponins during the course of hospital stay was reported; the pooled estimate was 34.2% (95% CI 26.2–42.6 %) (Figure S3). Seven studies (814 patients) enrolled only patients admitted to ICU [26, 29, 40, 42, 58, 62, 63], and the pooled prevalence of elevated troponins was 38.0% (95% CI 28.2–48.3%) (Figure S4).

### Elevated troponins and mortality

In 28 studies [13–15, 25–27, 31, 32, 34, 37, 38, 41, 43–46, 48, 49, 51, 52, 55, 57, 61, 65, 67, 68, 70, 71], data on the relationship between troponins (on admission or during hospital stay) and mortality of patients with covid-19 could be extracted. The risks of bias of these studies in assessing the prognostic value of troponins are summarised in Table S4. Many of the studies were at high risk of selection bias due to the lack of systematic troponin measurement and incomplete in-hospital follow-up. Besides, 10 studies did not adjust for relevant confounders (e.g. age, cardiovascular comorbidities). The results of studies that conducted multivariable analysis and the confounders adjusted for were listed in Table S5. In the following analyses, we focused only on studies that measured troponins on hospital admission, because troponin tests at this point of time might be useful for early risk stratification, whereas tests ordered during hospitalisation may have been a response to patients’ deteriorating conditions and thus would have more diagnostic but less predictive values.

**Fig. 2:**
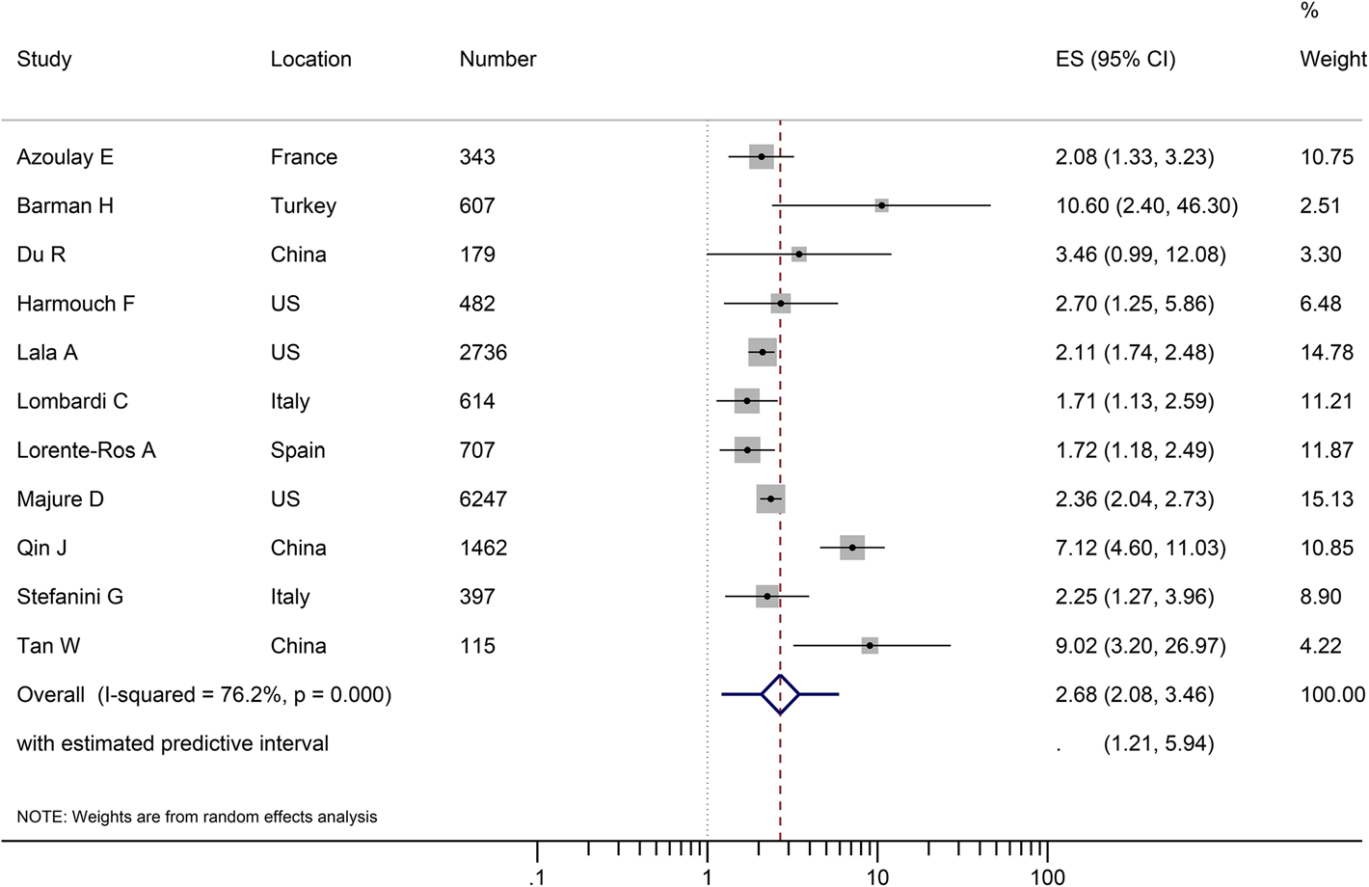
Forest plot showing the confounder-adjusted association between elevated troponins on hospital admission and mortality, quantified as risk ratio (RR) of death in patients with elevated troponins relative to those with non-elevated troponins. CI, confidence interval

We conducted a meta-analysis of 11 studies (13,889 patients) that reported multivariable-adjusted associations between admission troponins above the upper reference limit and mortality [13–15, 26, 27, 32, 37, 45, 46, 55, 67]. Elevated troponins on admission were associated with an increased risk of death (RR 2.68, 95% CI 2.08–3.46). There was substantial heterogeneity (*I*^2^ = 76.2%); the 95% prediction interval was wide (1.12–5.94) but did not include 1 (Fig. 2). Possible publication bias was found by the funnel plot (Figure S5a) but not confirmed by the Egger’s test (*P* = 0.203). We conducted a sensitivity analysis using the trim-and-fill method, which confirmed the stability of the association (RR 2.59, 95% CI 2.01–3.35) (Figure S5b). When we excluded 5 studies judged as having high risk of bias from meta-analysis, elevated troponins on admission remained a significant risk factor for subsequent death (RR 2.08, 95% CI 1.81–2.40), and the heterogeneity was eliminated (*I*^2^ = 0%). The results of subgroup analyses were shown in Table S6.

**Fig. 3:**
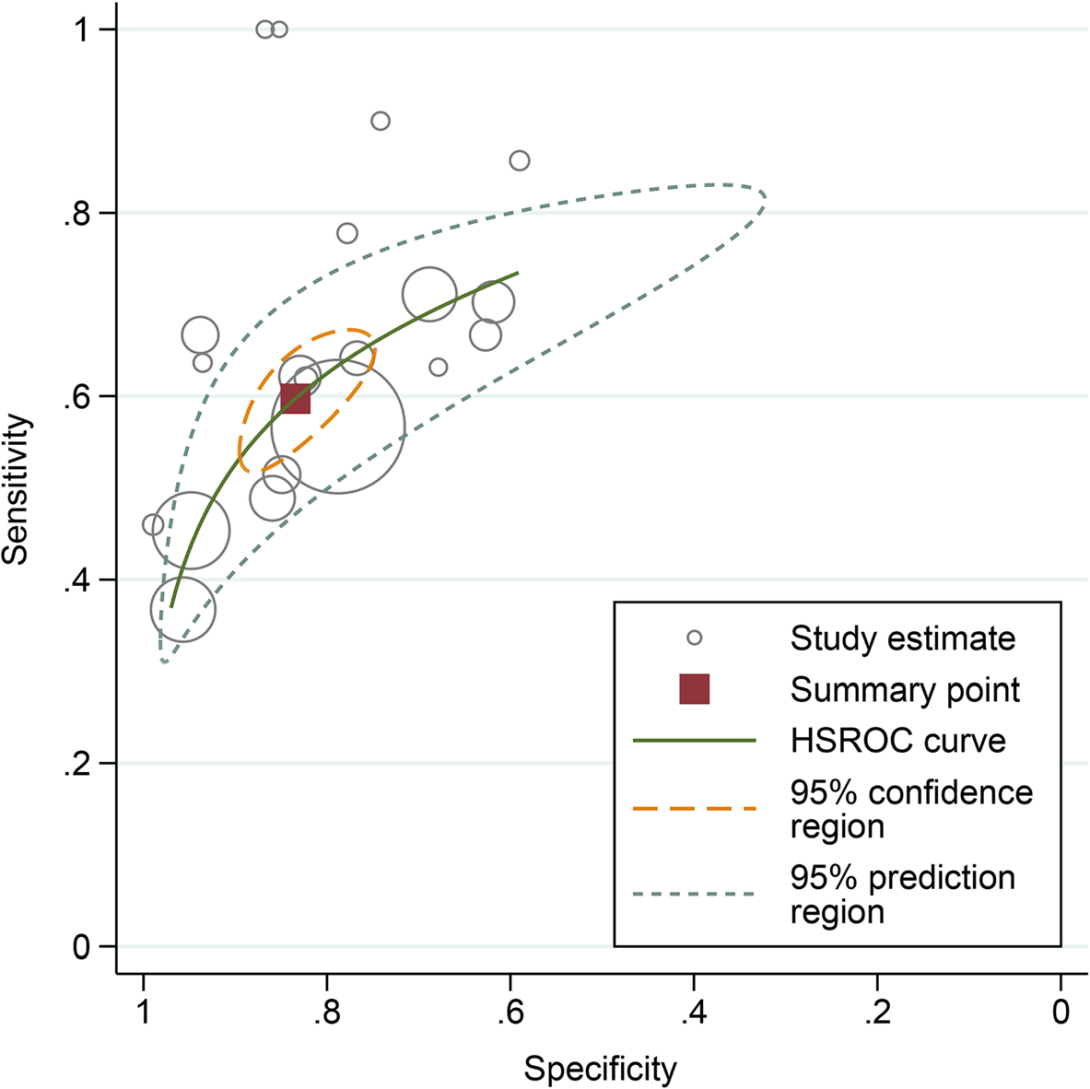
Performance of troponins on admission for predicting mortality. The brown square represents the summary operating point of the curve that summarises the prognostic performance of troponins (sensitivity 0.60, 95% CI 0.54–0.65; specificity 0.83, 95% CI 0.77–0.88; positive likelihood ratio 3.61, 95% CI 2.74–4.70; negative likelihood ratio 0.48, 95% CI 0.43–0.54). The area under the hierarchical summary receiver operating characteristic curve was 0.74, 95% CI 0.70–0.78.

**Fig. 4:**
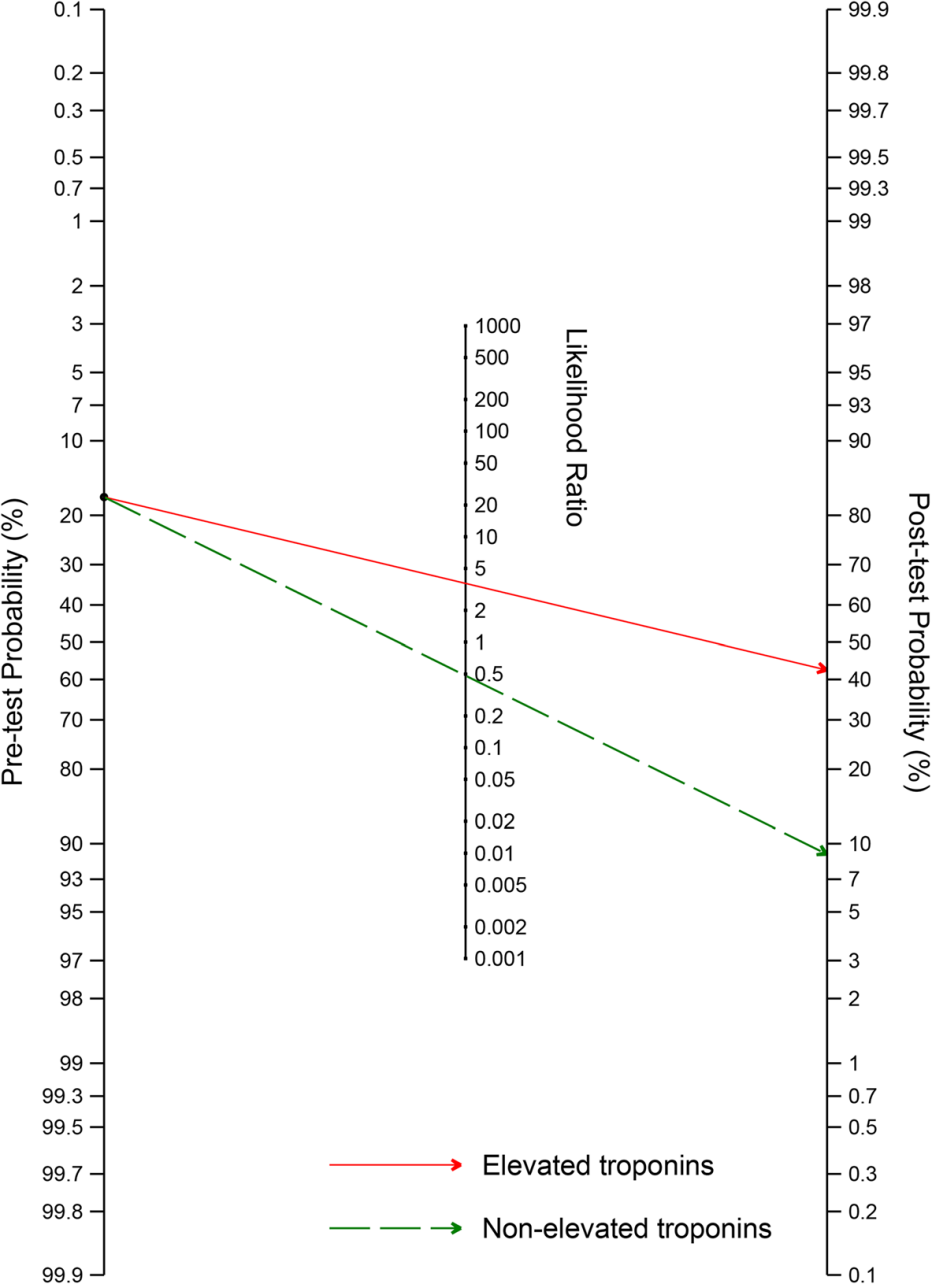
Fagan nomogram for calculation of post-test probability of death based on elevated (red) or non-elevated (green) troponins on admission. The Fagan nomogram is based on a pre-test probability of 17%, which is the pooled mortality estimate in 20 included studies, a positive likelihood ratio of 3.61, and a negative likelihood ratio of 0.48

To further characterise the predictive performance of troponins, we included 20 studies (15,488 patients) that reported the number of deaths in those admitted with elevated and non-elevated troponins in a bivariate meta-analysis [14, 15, 25, 27, 31, 32, 34, 37, 43–46, 51, 55, 57, 61, 65, 67, 70, 71]. The overall sensitivity of elevated troponins on admission for predicting death was 0.60 (95% CI 0.54–0.65); the specificity was 0.83 (0.77–0.88) (Fig. 3). The positive and negative likelihood ratios were 3.61 (2.74–4.70) and 0.48 (0.43–0.54), respectively. Considering a pre-test probability of death of 17% (the pooled estimate from the 20 included studies), the post-test probability of death for patients with elevated troponins on admission was approximately 42%, while that of patients with non-elevated troponins on admission was approximately 9% (Fig. 4).

## Discussion

The findings of this systematic review and meta-analysis demonstrated that elevated troponins are relatively common in patients hospitalised for covid-19 and appear to be independently associated with death.

Elevated troponins had a pooled prevalence of 20.8% in patients with covid-19 on hospital admission. The estimate appeared higher when the troponin concentrations during hospital stay was considered and in patients admitted to ICU. Our findings are consistent with those of previous studies showing that myocardial injury occurs frequently in patients with severe respiratory infections caused by other viruses or bacteria [72–74]. Common mechanisms of myocardial injury in these conditions may include oxygen supply-demand mismatch, systemic hyperinflammation, and microvascular dysfunction and thrombosis. Besides, recent pathological and imaging studies indicated that acute atherosclerotic plaque rupture, stress cardiomyopathy, direct cardiomyocytes damage by the SARS-CoV-2 virus and myocarditis may also be the causes of troponin elevation in some patients with covid-19 [75–78]. We observed significant heterogeneity in the prevalence of elevated troponins across individual studies, which is likely attributable to several factors. The demographics and burden of comorbidities of patient populations from different countries may be different. Besides, there are no universal criteria for hospital admission for covid-19 patients; the criteria may vary across different places and different phases of the disease spread. These resulted in considerable heterogeneity among study cohorts in baseline characteristics such as age, cardiovascular diseases and the severity of respiratory infection, which would produce widely varying frequencies of elevated troponins. Moreover, multiple troponin assays with different analytical sensitivities were used for assessing myocardial injury. Studies that used high-sensitivity assays may find higher prevalence of elevated troponins than those using less sensitive earlier-generation troponin assays. Despite the heterogeneity, our pooled estimate with a relatively narrow confidence interval represents a substantial minority of patients with elevated troponins on hospital admission, indicating an important involvement of the heart in severe forms of covid-19.

Similar to findings in patients with other severe respiratory illnesses, elevated troponins appeared to have prognostic implications for patients admitted for covid-19. Some recent systematic reviews and meta-analyses have reported on the associations between elevated troponins and adverse outcomes of covid-19 [5–8, 79, 80]. However, there are several limitations to these analyses, including not accounting for the time point of troponin measurements and the cut-off threshold for troponin elevation, the variability in outcome definitions, the lack of adjustment for confounders and the inclusion of overlapping cohorts in analysis. In this study, we addressed the predictive value of troponin by focusing on troponin measured on hospital admission and on the hard outcome of death. We found that admission troponin concentrations higher than the upper reference limit were associated with a more than twofold risk of death in multivariable analyses. The stability of this association was supported by various sensitivity and subgroup analyses. Thus, troponin testing may provide prognostic information independent of other routinely assessed demographic and clinical factors for covid-19 patients early on patient admission, so that it might help clinicians in triage decision-making. Our results suggested that measurement of troponin levels at the time of hospital admission for covid-19 might be included in the diagnostic workup to identify patients at increased risk of worse outcome and those who may require higher level of surveillance and more intensive treatment.

In addition, our bivariate analysis suggested that troponin may be especially helpful to identify patients at high mortality risk when it is elevated. However, the prognosis of patients with normal troponin levels was still worrisome (mortality of ~ 9%). This is not surprising because injuries in other organ systems during the course of disease are also important factors of death [81]. Thus, the detection of a normal troponin level at hospital admission may not be considered as a sign of very low mortality risk or criteria for early discharge. The combination of troponin with other clinical information might achieve more accurate prognostication than either alone [45].

While higher mortality rates of patients with elevated troponins were consistently reported by the included studies, only two studies ascertained the mechanisms of death of patients [14, 15]. Interestingly, there was no significant difference in causes of death between patients with or without elevated troponin who deceased. It might be hypothesised that elevated troponins reflect the severity of involvement of different organs and tissues in covid-19 patients and may predict higher mortality of both cardiovascular and non-cardiovascular causes.

We acknowledge several limitations to our meta-analysis. There is significant heterogeneity in the prevalence of elevated troponins on admission and the strength of their association with mortality, presumably reflecting differing patient background, troponin assays and therapeutic practices among study centres. In addition, many of the included studies did not measure troponin systematically for all patients on their admission, and the indications for troponin measurement were poorly reported. Selective troponin sampling may have resulted in a systematic overestimation of the prevalence of elevated troponins in these studies. However, our pooled analysis of 19 studies at low risk of bias (studies that measured troponin concentrations on admission in > 90% of patients) yielded similar prevalence estimate. On the other hand, patients that did not received troponin measurement tended to have milder illness, lower prevalence of myocardial injury and lower risks of subsequent death [49]. Thus, their exclusion might have biassed the observed strength of association between elevated troponins and death toward a smaller magnitude. Overall, while we cannot be certain about the accuracy of estimates for the prevalence of elevated troponins and the associated risk of death, we believe that important inferences can still be made. Across a heterogeneous group of patients hospitalised for covid-19, myocardial injury reflected by troponin concentrations above the upper reference limit was relatively common and did consistently correlate with excess risk of death.

In this study, despite that we evaluated the association between elevated troponins and death based on multivariable analyses, our result may still be subject to residual confounding. Baseline comorbidities such as cardiovascular and kidney diseases are both strongly associated with higher troponin concentrations and are independent risk factors for mortality of covid-19 [82, 83], but they were not fully adjusted for in some of the included studies. However, dissecting the relative contributions of entwined conditions of pre-existing chronic myocardial injury, new-onset acute myocardial injury and reduced troponin clearance caused by renal impairment may be impossible, even if individual patient data from original studies were available. Thus, we could not address the question whether covid-19 infection-related myocardial injury directly influences the survival of patients. However, this limitation should not detract from the potential value of elevated troponins as a marker for early identification of covid-19 patients at high risk of death.

Other limitations of this study included the fact that we could not evaluate the utility of serial troponin testing during the first few days after admission in the identification of and risk assessment for patients with myocardial injury, because single troponin measurements on admission were reported in most studies. In addition, we were not able to evaluate the long-term impact of elevated troponins on admission or during hospital stay on the cardiovascular health of covid-19 survivors. Given the limitations of available evidence, the justification of measuring troponin as a prognostic tool for patients hospitalised for covid-19 warrants further investigation. Ongoing registries that systematically collect cardiovascular data in covid-19 patients, such as the CAPACITY-COVID [84], will hopefully contribute to a better understanding of the implications of troponin measurements in patients with covid-19.

## Conclusion

The present meta-analysis suggests that among patients hospitalised for covid-19, admission troponin concentrations above the upper reference limit are common and are predictive for subsequent death. Clinically, the presence of elevated troponins on admission may facilitate risk stratification by enabling early identification of patients at high mortality risk. Large prospective studies with systematic troponin sampling and adequate follow-up are needed to validate the prognostic implications of elevated troponins for patients admitted for covid-19.

## Supplementary Information

**Additional file 1: Table S1.** Preferred Reporting Items for Systematic Reviews and Meta-Analyses (PRISMA) checklist. **Table S2.** Literature search strategy (PubMed as example). **Table S3.** Risk of bias assessment for studies on the prevalence of elevated troponin in patients hospitalised for covid-19. **Table S4.** Quality in Prognostic Studies (QUIPS) risk of bias assessment for studies on the association between elevated troponin and mortality. **Table S5.** Multivariable-adjusted association between elevated troponin and mortality in patients hospitalised for covid-19. **Table S6.** Subgroup analyses on the prognostic value of elevated troponins on admission for predicting death. **Table S7.** Covid-19 severity of illness classification. **Figure S1.** Pooled prevalence of elevated troponins on hospital admission. **Figure S2.** Funnel plot for assessing publication bias in the prevalence of elevated troponins on hospital admission. **Figure S3.** Pooled prevalence of elevated troponins during hospital stay. **Figure S4.** Pooled prevalence of elevated troponins in patients admitted to intensive care unit. **Figure S5.** (a) Funnel plot for assessing publication bias in the association between elevated admission troponins and mortality risk. (b) Funnel plot after applying the trim-and-fill method.

## Data Availability

The datasets used and/or analysed during the current study are available from the corresponding author on reasonable request.

## Abbreviations

Covid-19: Coronavirus disease 2019
SARS-CoV2: Severe acute respiratory syndrome coronavirus-2
CHARMS: CHecklist for critical Appraisal and data extraction for systematic Reviews of prediction Modelling Studies
PRISMA: Preferred Reporting Items for Systematic Reviews and Meta-Analyses
CI: Confidence interval
QUIPS: Quality in Prognosis Studies
RR: Risk ratio
PI: Prediction interval
