# Supplementary material for: Prevalence and prognostic value of elevated troponins in patients hospitalised for coronavirus disease 2019: a systematic review and meta-analysis
Source: J Intensive Care. 2020 Nov 23;8:88. doi: 10.1186/s40560-020-00508-6 (PMC7682759; doi:10.1186/s40560-020-00508-6)

**Prevalence and Prognostic Value of Elevated Troponin in Patients Hospitalised for Coronavirus Disease 2019: A Systematic Review and Meta-analysis**

Bing-Cheng Zhao, Wei-Feng Liu, Shao-Hui Lei, Bo-Wei Zhou, Xiao Yang, Tong-Yi Huang, Qi-Wen Deng, Miao Xu, Cai Li, and Ke-Xuan Liu

**Supplementary materials**

Table S1. Preferred Reporting Items for Systematic Reviews and Meta-Analyses (PRISMA) checklist

Table S2. Literature search strategy (PubMed as example)

Table S3. Risk of bias assessment for studies on the prevalence of elevated troponin in patients hospitalised for covid-19

Table S4. Quality in Prognostic Studies (QUIPS) risk of bias assessment for studies on the association between elevated troponin and mortality

Table S5. Multivariable-adjusted association between elevated troponin and mortality in patients hospitalised for covid-19

Table S6. Subgroup analyses on the prognostic value of elevated troponins on admission for predicting death

Table S7. Covid-19 severity of illness classification

Figure S1. Pooled prevalence of elevated troponins on hospital admission

Figure S2. Funnel plot for assessing publication bias in the prevalence of elevated troponins on hospital admission

Figure S3. Pooled prevalence of elevated troponins during hospital stay

Figure S4. Pooled prevalence of elevated troponins in patients admitted to intensive care unit

Figure S5 (a) Funnel plot for assessing publication bias in the association between elevated admission troponins and mortality risk. (b) Funnel plot after applying the trim-and-fill method.

**Table S1. Preferred Reporting Items for Systematic Reviews and Meta-Analyses (PRISMA) checklist**

| **Section/topic** | **#** | **Checklist item** | **Reported on page #** |
| --- | --- | --- | --- |
| **Title** | | |  |
| Title | 1 | Identify the report as a systematic review, meta-analysis, or both. | P1 |
| **Abstract** | | |  |
| Structured summary | 2 | Provide a structured summary including, as applicable: background; objectives; data sources; study eligibility criteria, participants, and interventions; study appraisal and synthesis methods; results; limitations; conclusions and implications of key findings; systematic review registration number. | P2 |
| **Introduction** | | |  |
| Rationale | 3 | Describe the rationale for the review in the context of what is already known. | P4 |
| Objectives | 4 | Provide an explicit statement of questions being addressed with reference to participants, interventions, comparisons, outcomes, and study design (PICOS). | P5 |
| **Methods** | | |  |
| Protocol and registration | 5 | Indicate if a review protocol exists, if and where it can be accessed (e.g., Web address), and, if available, provide registration information including registration number. | P5 |
| Eligibility criteria | 6 | Specify study characteristics (e.g., PICOS, length of follow-up) and report characteristics (e.g., years considered, language, publication status) used as criteria for eligibility, giving rationale. | P5-6 |
| Information sources | 7 | Describe all information sources (e.g., databases with dates of coverage, contact with study authors to identify additional studies) in the search and date last searched. | P5 |
| Search | 8 | Present full electronic search strategy for at least one database, including any limits used, such that it could be repeated. | P5, Table S2 |
| Study selection | 9 | State the process for selecting studies (i.e., screening, eligibility, included in systematic review, and, if applicable, included in the meta-analysis). | P5-6 |
| Data collection process | 10 | Describe method of data extraction from reports (e.g., piloted forms, independently, in duplicate) and any processes for obtaining and confirming data from investigators. | P6 |
| Data items | 11 | List and define all variables for which data were sought (e.g., PICOS, funding sources) and any assumptions and simplifications made. | P6 |
| Risk of bias in individual studies | 12 | Describe methods used for assessing risk of bias of individual studies (including specification of whether this was done at the study or outcome level), and how this information is to be used in any data synthesis. | P7 |
| Summary measures | 13 | State the principal summary measures (e.g., risk ratio, difference in means). | P7-9 |
| Synthesis of results | 14 | Describe the methods of handling data and combining results of studies, if done, including measures of consistency (e.g., I^2^) for each meta-analysis. | P7-9 |
| Risk of bias across studies | 15 | Specify any assessment of risk of bias that may affect the cumulative evidence (e.g., publication bias, selective reporting within studies). | P8 |
| Additional analyses | 16 | Describe methods of additional analyses (e.g., sensitivity or subgroup analyses, meta-regression), if done, indicating which were pre-specified. | P8 |
| **Results** |  |  |  |
| Study selection | 17 | Give numbers of studies screened, assessed for eligibility, and included in the review, with reasons for exclusions at each stage, ideally with a flow diagram. | P9, Fig 1 |
| Study characteristics | 18 | For each study, present characteristics for which data were extracted (e.g., study size, PICOS, follow-up period) and provide the citations. | P9, Table 1 & 2 |
| Risk of bias within studies | 19 | Present data on risk of bias of each study and, if available, any outcome level assessment (see item 12). | P10-11, Table S3 & S4 |
| Results of individual studies | 20 | For all outcomes considered (benefits or harms), present, for each study: (a) simple summary data for each intervention group (b) effect estimates and confidence intervals, ideally with a forest plot. | P9-11; Fig 2, S1, S3, S4; Table 2, S5 |
| Synthesis of results | 21 | Present results of each meta-analysis done, including confidence intervals and measures of consistency. | P9-11; Fig 2, 3, S1, S3, S4 |
| Risk of bias across studies | 22 | Present results of any assessment of risk of bias across studies (see Item 15). | P10-11; Fig S2, S5 |
| Additional analysis | 23 | Give results of additional analyses, if done (e.g., sensitivity or subgroup analyses, meta-regression [see Item 16]). | P10-12; Table S6 |
| **Discussion** |  |  |  |
| Summary of evidence | 24 | Summarize the main findings including the strength of evidence for each main outcome; consider their relevance to key groups (e.g., healthcare providers, users, and policy makers). | P12-15 |
| Limitations | 25 | Discuss limitations at study and outcome level (e.g., risk of bias), and at review-level (e.g., incomplete retrieval of identified research, reporting bias). | P15-17 |
| Conclusions | 26 | Provide a general interpretation of the results in the context of other evidence, and implications for future research. | P17-18 |
| **Funding** |  |  |  |
| Funding | 27 | Describe sources of funding for the systematic review and other support (e.g., supply of data); role of funders for the systematic review. | P18 |

**Table S2. Literature search strategy (PubMed as example)**

| **Search** | **Query** | **Items found** |
| --- | --- | --- |
| #1 | ("2019 nCoV" OR "2019nCoV" OR "2019-nCoV" OR "COVID 19" OR "COVID19" OR "COVID-19" OR "new coronavirus" OR "novel coronavirus" OR (Wuhan AND coronavirus) OR (Wuhan AND pneumonia) OR "SARS-CoV" OR "SARS-CoV-2" OR "SARS CoV-2") | 64780 |
| #2 | ("troponin" OR "cardiac" OR "myocardial" OR "TnI" OR "TnT" OR "cardiovascular" OR "heart") | 2,320,802 |
| #3 | #1 AND #2 AND ("2019/12/01"[Date - Publication] : "3000"[Date - Publication]) | 5,571 |

**Table S3. Risk of bias assessment for studies on the prevalence of elevated troponin in patients hospitalised for covid-19**

| **Study author** | **Question 1** | **Question 2** | **Question 3** | **Question 4** | **Question 5** | **Question 6** | **Overall risk of bias** |
| --- | --- | --- | --- | --- | --- | --- | --- |
| Arcari L, et al | Yes | Yes | Yes | Yes | No | Yes | High |
| Azoulay E, et al | No | Yes | Yes | Yes | No | Yes | High |
| Barman H, et al | Yes | No | Yes | Yes | Yes | Yes | High |
| Bhatla A, et al | Yes | No | Yes | Yes | Yes | Yes | High |
| Bhatraju P, et al | No | No | Yes | No | No | Yes | High |
| Buckner F, et al | Yes | No | Yes | No | No | Yes | High |
| Cipriani A, et al | Yes | No | Yes | Yes | Yes | Yes | High |
| Du R, et al | Yes | Yes | Yes | Yes | Yes | Yes | Low |
| Ferguson J, et al | Yes | No | Yes | Yes | Yes | Yes | High |
| Franks C, et al | Yes | No | Yes | Yes | Yes | Yes | High |
| Gottlieb M, et al | Yes | No | Yes | Yes | Yes | Yes | High |
| Goyal P, et al | Yes | No | Yes | Yes | Yes | Yes | High |
| Harmouch F, et al | Yes | No | Yes | No | No | Yes | High |
| He H, et al | No | Yes | Yes | No | No | Yes | High |
| Heberto A, et al | Yes | Yes | Yes | Yes | Yes | Yes | Low |
| Hu L, et al | Yes | Yes | Yes | Yes | Yes | Yes | Low |
| Huang M, et al | Yes | Yes | Yes | Yes | Yes | Yes | Low |
| Karbalai S, et al | Yes | Yes | Yes | No | No | Yes | High |
| Lala A, et al | Yes | No | Yes | Yes | Yes | Yes | High |
| Lazzeri C, et al | No | Yes | Yes | Yes | Yes | Yes | High |
| Li C, et al | Yes | Yes | Yes | Yes | Yes | Yes | Low |
| Li C, et al | Yes |  | Yes | Yes | Yes | Yes | High |
| Lombardi C, et al | Yes | Yes | Yes | Yes | No | Yes | High |
| Lorente-Ros A, et al | Yes | Yes | Yes | Yes | Yes | Yes | Low |
| Lu S, et al | No | No | Yes | No | No | Yes | High |
| Ma K, et al | Yes | Yes | Yes | No | No | Yes | High |
| Majure D, et al | Yes | No | Yes | Yes | No | Yes | High |
| Mejía-Vilet J, et al | Yes | Yes | Yes | Yes | Yes | Yes | Low |
| Nguyen A, et al | Yes | Yes | Yes | Yes | Yes | Yes | Low |
| Nie S, et al | Yes | No | Yes | No | No | Yes | High |
| Price-Haywood, et al | Yes | No | Yes | Yes | Yes | Yes | High |
| Qi D, et al | Yes | No | Yes | Yes | Yes | Yes | High |
| Qin J, et al | Yes | No | Yes | Yes | No | Yes | High |
| Raad M, et al | Yes | Yes | Yes | Yes | Yes | Yes | Low |
| Shah P, et al | Yes | No | Yes | No | No | Yes | High |
| Shah S, et al | Yes | No | Yes | No | No | Yes | High |
| Shen Y, et al | Yes | Yes | Yes | Yes | Yes | Yes | Low |
| Stefanini G, et al | Yes | Yes | Yes | Yes | Yes | Yes | Low |
| Szekely Y, et al | Yes | Yes | Yes | Yes | Yes | Yes | Low |
| Tan W, et al | Yes | Yes | Yes | Yes | Yes | Yes | Low |
| van den Heuvel F, et al | Yes | Yes | Yes | No | No | Yes | High |
| Wei J, et al | Yes | Yes | Yes | Yes | Yes | Yes | Low |
| Xu W, et al | Yes | Yes | Yes | Yes | Yes | Yes | Low |
| Yang S, et al | Yes | Yes | Yes | Yes | Yes | Yes | Low |
| Yu Y, et al | No | Yes | Yes | No | No | Yes | High |
| Zeng J, et al | Yes | No | Yes | Yes | Yes | Yes | High |
| Zhang J, et al | Yes | Yes | Yes | Yes | Yes | Yes | Low |
| Zhao X, et al | Yes | Yes | Yes | Yes | Yes | Yes | Low |
| Zhou F, et al | Yes | No | Yes | Yes | Yes | Yes | High |

**Question 1.** Is the sampling frame a true or close representation of the target population?

No if the sampling frame is not representative of average patients hospitalised for covid-19 pneumonia. For example, only patients with certain comorbidities or patients in ICU were sampled.

**Question 2.** Is an unselected (random/consecutive) sample of patients enrolled for troponin measurement?

No if less than 90% of patients received troponin measurement, or the researchers did an analysis and found significant differences in baseline characteristics between patients who received troponin measurement and those who did not, or such an analysis was not carried out.

**Question 3.** Is an acceptable definition for elevated troponins used in the study?

No if the laboratory-specific upper reference limit was not used as the cut-off for elevated troponins.

**Question 4.** Are the timing and assay of troponin measurement valid?

No if troponin measurements using approved commercial assays were not conducted within 72 hours of admission to hospital.

**Question 5.** Is same mode of data collection used for all patients?

No if the timing of troponin measurement was not the same for all patients or different troponin assays were used.

**Question 6.** Were the numerator and denominator for calculating the prevalence of elevated troponin appropriate?

No if denominator was not the number of patients who received troponin measurement, but the total number of patients enrolled.

**Table S4. Quality in Prognostic Studies (QUIPS) risk of bias assessment for studies on the association between elevated troponin and mortality**

| **Study author** | **Study participation** | **Study attrition** | **Prognostic factor (elevated troponin) measurement** | **Outcome (death) assessment** | **Study confounding** | **Statistical analysis and reporting** | **Overall risk of bias** |
| --- | --- | --- | --- | --- | --- | --- | --- |
| Arcari L, et al | Yes | No ^c^ | Yes | Yes | No ^g^ | Yes | High |
| Azoulay E, et al | Yes | Partly ^d^ | Yes | Yes | Yes | Yes | Moderate |
| Barman H, et al | No ^a^ | No ^c^ | Yes | Yes | Yes | Yes | High |
| Cipriani A, et al | Partly ^b^ | Yes | Yes | Yes | No ^g^ | Yes | High |
| Du R, et al | Yes | Yes | Yes | Yes | Yes | Yes | Low |
| Franks C, et al | No ^a^ | No ^c^ | Yes | Yes | No ^g^ | Partly ^i^ | High |
| Harmouch F, et al | Partly ^b^ | Yes | Yes | Yes | Yes | Partly ^i^ | Moderate |
| Heberto A, et al | Yes | Yes | Yes | Yes | Yes | Partly ^j^ | Moderate |
| Karbalai S, et al | Yes | Yes | No ^e^ | Yes | Yes | Yes | High |
| Lala A, et al | Partly ^b^ | Partly ^d^ | Yes | Yes | Yes | Yes | Moderate |
| Li C, et al | Yes | Partly ^d^ | Yes | Yes | No ^g^ | Yes | High |
| Li C, et al | No ^a^ | No ^c^ | Yes | Yes | Yes | Partly ^i^ | High |
| Lombardi C, et al | Yes | No ^c^ | Yes | Yes | Yes | Yes | High |
| Lorente-Ros A, et al | Yes | Yes | Yes | Yes | Partly ^h^ | Yes | Moderate |
| Majure D, et al | No ^a^ | Yes | Yes | Yes | Yes | Partly ^i^ | High |
| Nguyen A, et al | Yes | Yes | Yes | Yes | No | Yes | High |
| Nie S, et al | No ^a^ | Yes | No ^e^ | Yes | Yes | Yes | High |
| Petrilli C, et al | Yes | Yes | Unclear ^f^ | Yes | Yes | Yes | Moderate |
| Qin J, et al | No ^a^ | No ^c^ | Yes | Yes | Yes | Yes | High |
| Raad M, et al | Yes | No ^c^ | Yes | Yes | No ^g^ | Yes | High |
| Shah P, et al | No ^a^ | No ^c^ | No ^e^ | Yes | Yes | Partly ^j^ | High |
| Stefanini G, et al | Yes | Yes | Yes | Yes | Partly ^h^ | Yes | Moderate |
| Tan W, et al | Yes | No ^c^ | Yes | Yes | Partly ^h^ | Partly ^j^ | High |
| Wei J, et al | Yes | No ^c^ | Yes | Yes | No ^g^ | Yes | High |
| Woo S, et al | No ^a^ | No ^c^ | Yes | Yes | Partly ^h^ | Yes | High |
| Yang S, et al | Yes | No ^c^ | Yes | Yes | No ^g^ | Yes | High |
| Zhang J, et al | Yes | No ^c^ | Yes | Yes | No ^g^ | Yes | High |
| Zhou F, et al | No ^a^ | No ^c^ | Yes | Yes | No ^g^ | Yes | High |

^a^ Less than 80% or unclear percentage of enrolled patients had troponin measurement.

^b^ 80-90% of enrolled patients had troponin measurement.

^c^ Less than 60% or unclear percentage of patients completed in-hospital follow-up.

^d^ 60-80% of patients completed in-hospital follow-up.

^e^ The decision of measuring troponin or not may have been influenced by the severity of disease.

^f^ It is unclear whether the laboratory-specific upper limit of normal was used as the cut-off for troponin elevation.

^g^ No confounder adjustment was performed.

^h^ Multivariable analysis was done but important confounders (age and cardiovascular comorbidities) were not fully adjusted.

^j^ The regression model was at risk of overfitting due to the small number of outcomes.

^i^ Time-to-event analysis was not used for studies containing patients who neither recovered nor died within the follow-up period.

**Table S5. Multivariable-adjusted association between elevated troponin and mortality in patients hospitalised for covid-19**

| **Study** | **Adjusted OR/HR (95% CI) ^a^** | **Covariates adjusted in multivariable model** |
| --- | --- | --- |
| **Elevated troponin at the time of admission** | | |
| Azoulay E, et al | HR 2.08 (1.33-3.23) | Age, asthma, diabetes, COPD, hypertension, immunosuppression, time from viral symptom onset to intensive care unit admission, acute kidney injury |
| Barman H, et al | HR 10.6 (2.4-46.3) | Age, hypertension, CAD, COPD, creatinine, uric acid, glucose, CRP, d-dimer |
| Du R, et al | OR 4.08 (1.17-14.25) | Age, hypertension, cardiovascular or cerebrovascular disease, dyspnea, fatigue, sputum production, headache, leukocyte count, neutrophil count, CD3+ CD8+ T cell count, myoglobin, creatinine, d-dimer, PaO_2_ |
| Harmouch F, et al | OR 3.22 (1.49-6.99) | Age, CKD, vascular disease, lymphopenia, elevated ferritin |
| Lala A, et al | Troponin ≤ URL as reference: troponin 1-3 URL, HR 1.75 (1.37-2.24); troponin >3 URL, HR 3.03 (2.42-3.80) | Age, gender, race, ethnicity, CAD, atrial fibrillation, heart failure, hypertension, CKD, diabetes, in-hospital intubation |
| Li C, et al | OR per standard deviation increase in troponin 1.18 (1.00-1.40) | Age, sex, hypertension, diabetes, CAD, arrhythmia, cancer, stroke, heart failure, COPD, CKD, interleukin-6, d-dimer |
| Lombardi, et al | HR 1.71 (1.13-2.59) | Age, sex, SpO_2_, CRP, estimated glomerular filtration rate, PaO_2_/FiO_2_ ratio, CAD, heart failure, atrial fibrillation, COPD, hypertension, CKD |
| Lorente-Ros A, et al | HR 1.72 (1.18-2.49) | Age, creatinine, CRP |
| Majure D, et al | Troponin ≤ URL as reference: troponin 1-3 URL, OR 2.06 (1.68-2.53); troponin >3 URL OR 4.51 (3.66-5.54) | Age, sex, race, ethnicity, hypertension, CAD, heart failure, peripheral vascular disease, COPD, diabetes, use of ACEI/ARB, alanine aminotransferase, serum creatinine |
| Petrilli C, et al | Troponin <0.1 ng/mL as reference:  0.1-1 ng/mL, HR 1.48 (1.19-1.84);  >1 ng/mL, HR 2.12 (1.39-3.22) | Week of admission, age, sex, race/ethnicity, smoking status, BMI, CAD, heart failure, hyperlipidemia, hypertension, diabetes, asthma or COPD, CKD, cancer, temperature, oxygen saturation, lymphocyte count, creatinine, CRP, d-dimer, ferritin, procalcitonin |
| Qin J, et al | HR 7.12 (4.60,11.03) | Age, sex, diabetes, hypertension, CAD, cerebrovascular disease |
| Stefanini G, et al | HR 2.25 (1.27-3.96) | Age, estimated glomerular filtration rate, PaO_2_/FiO_2_ ratio, pulmonary artery diameter, ACEI/ARB use |
| Tan W, et al ^b^ | HR 9.02 (3.20-26.97) | Procalcitonin, neutrophil percentage, neutrophil/lymphocyte ratio, eosinophil percentage, aspartate aminotransferase, total bilirubin, urea |
| Woo S, et al | OR 1.87 (1.04-3.38) | Age, sex, dyspnea, diabetes, CRP, aspartate aminotransferase, d-dimer |
| **Elevated troponin during the course of hospitalisation** | | |
| Heberto A, et al | OR 3.76 (1.31-10.84) | Age, sex, hypertension, diabetes, over weight or obesity, NT-proBNP, ferritin, hsCRP, lymphocyte count, d-dimer, localized T-wave inversion, septic shock, acute respiratory distress syndrome, hemodialysis, hydroxychloroquine/ azithromycin, steroids, anticoagulant therapy |
| Karbalai S, et al | HR 1.81 (1.09-3.02) | Age, diabetes, cardiovascular disease (hypertension, CAD, or congestive heart failure), malignancy, CKD, cerebrovascular accident, use of ACEI/ARB, acute respiratory distress syndrome, acute kidney injury |
| Nie S, et al ^b^ | HR per 2-fold increase in troponin concentration 1.92 (1.41-2.59) | Age, sex, comorbidity (hypertension, diabetes, CAD, arrythmia, heart failure, or cerebrovascular disease), body temperature, SpO_2_, disease severity, lymphocyte count, CRP, d-dimer |
| Raad M, et al | Troponin <18 ng/L as reference: 19-99 ng/L, 3.0 (1.5-6.0); ≥100 ng/L, 7.7 (3.7-16.0) | Age ≥ 65, gender, BMI, hypertension, CAD, heart failure, atrial fibrillation/flutter, cerebrovascular disease, immunosuppressed state, COPD, CKD, cirrhosis. peak levels of lactate dehydrogenase, CRP, ferritin and d-dimer, acute respiratory distress syndrome, acute kidney injury |
| Shah P, et al | OR 4.45 (1.78-11.14) | age, gender, race, BMI, comorbidities, presenting illness severity, d-dimer, lactate dehydrogenase, procalcitonin, ferritin, CRP |

ACEI/ARB, angiotensin converting enzyme inhibitors/angiotensin II receptor blockers; BMI, body mass index; CAD, coronary artery disease; CKD, chronic kidney disease; COPD, chronic obstructive pulmonary disease; FiO_2_, fraction of inspired oxygen; HR, hazard ratio; hsCRP, high-sensitivity C-reactive protein; NT-proBNP, N-terminal pro-B-type natriuretic peptide; OR, odds ratio; PaO_2_, partial pressure of arterial blood; SpO_2_, peripheral capillary oxygen saturation, URL, upper reference limit.

^a^ Troponin concentrations are dichotomised at the laboratory-specific upper reference limit, excepted otherwise stated.

^b^ These studies used an overlapping cohort.

**Table S6. Subgroup analyses on the prognostic value of elevated troponins on admission for predicting death**

|  | **Adjusted risk ratio (95% CI)** | **Sensitivity**  **(95% CI)** | **Specificity**  **(95% CI)** | **Positive likelihood ratio (95% CI)** | **Negative likelihood ratio (95% CI)** |
| --- | --- | --- | --- | --- | --- |
| **Subgroup 1** |  |  |  |  |  |
| Troponin T | — | — | — | — | — |
| Troponin I | 3.37 (2.15-5.30) (8) | 0.60 (0.53-0.66) (17) | 0.85 (0.79-0.90) (17) | 4.04 (3.04-5.37) (17) | 0.47 (0.42-0.54) (17) |
| **Subgroup 2** |  |  |  |  |  |
| High-sensitivity assays | 3.49 (1.64-7.40) (4) | 0.61 (0.54-0.67) (12) | 0.83 (0.76-0.89) (12) | 3.66 (2.63-5.09) (12) | 0.47 (0.42-0.53) (12) |
| Contemporary assays | 2.16 (1.82-2.56) (3) | 0.66 (0.51-0.79) (4) | 0.84 (0.72-0.91) (4) | 4.03 (2.47-6.60) (4) | 0.40 (0.28-0.60) (4) |
| **Subgroup 3** |  |  |  |  |  |
| Studies in China | 6.86 (4.67-10.08) (3) | 0.59 (0.47-0.70) (9) | 0.91 (0.87-0.94) (9) | 6.65 (4.94-8.94) (9) | 0.45 (0.35-0.59) (9) |
| Studies outside China | 2.16 (1.89-2.46) (8) | 0.64 (0.57-0.70) (11) | 0.74 (0.68-0.79) (11) | 2.43 (2.10-2.81) (11) | 0.49 (0.44-0.56) (11) |
| **Subgroup 4** |  |  |  |  |  |
| Peer-reviewed | 2.53 (1.97-3.23) (10) | 0.59 (0.52-0.65) (16) | 0.83 (0.76-0.88) (16) | 3.44 (2.62-4.51) (16) | 0.50 (0.45-0.55) (16) |
| Preprint without review | 9.02 (3.11-26.19) (1) | 0.68 (0.58-0.76) (4) | 0.86 (0.70-0.94) (4) | 4.68 (2.09-10.49) (4) | 0.38 (0.28-0.51) (4) |
| **Subgroup 5** |  |  |  |  |  |
| High risk of bias | 4.08 (2.19-7.62) (5) | 0.59 (0.52-0.67) (13) | 0.86 (0.77-0.91) (13) | 4.09 (2.73-6.13) (13) | 0.48 (0.41-0.55) (13) |
| Moderate-low risk of bias | 2.08 (1.81-2.40) (6) | 0.60 (0.52-0.68) (7) | 0.79 (0.73-0.84) (7) | 2.91 (2.39-3.55) (7) | 0.50 (0.42-0.59) (7) |
| **Subgroup 6** |  |  |  |  |  |
| Sample size ≤500 | 2.79 (1.83-4.24) (5) | 0.66 (0.58-0.73) (13) | 0.83 (0.75-0.89) (13) | 3.93 (2.69-5.75) (13) | 0.41 (0.34-0.50) (13) |
| Sample size >500 | 2.63 (1.89-3.67) (6) | 0.55 (0.46-0.64) (7) | 0.85 (0.74-0.92) (7) | 3.61 (2.34-5.58) (7) | 0.53 (0.47-0.59) (7) |

The number in brackets after the pooled estimates is the number of studies included in the corresponding subgroup.

CI, confidence interval.

**Table S7 Covid-19 severity of illness classification**

| **Categories** | **Definitions** |
| --- | --- |
| Mild Illness | Individuals who have any of the various signs and symptoms of COVID-19 (e.g., fever, cough, sore throat, malaise, headache, muscle pain, nausea, vomiting, diarrhea, loss of taste and smell) but who do not have shortness of breath, dyspnea, or abnormal chest imaging. |
| Moderate Illness | Individuals who show evidence of lower respiratory disease during clinical assessment or imaging and who have saturation of oxygen (SpO2) ≥94% on room air at sea level. |
| Severe Illness | Individuals who have SpO2 <94% on room air at sea level, a ratio of arterial partial pressure of oxygen to fraction of inspired oxygen (PaO2/FiO2) <300 mmHg, respiratory frequency >30 breaths per minute, or lung infiltrates >50%. |
| Critical Illness | Individuals who have respiratory failure, septic shock, and/or multiple organ dysfunction. |

**Figure S1. Pooled prevalence of elevated troponins on hospital admission**


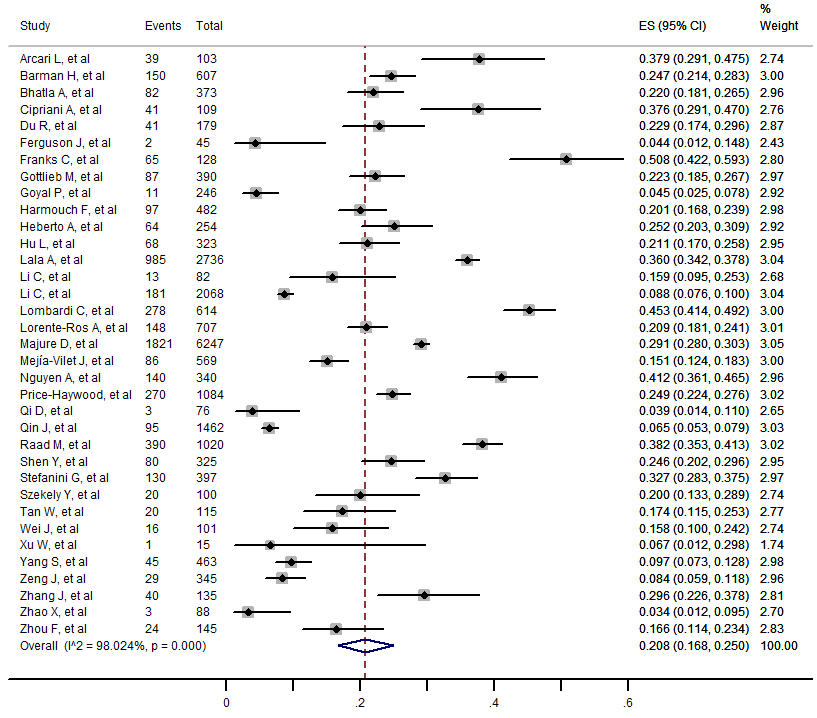


**Figure S2. Funnel plot for assessing publication bias in the prevalence of elevated troponins on hospital admission**


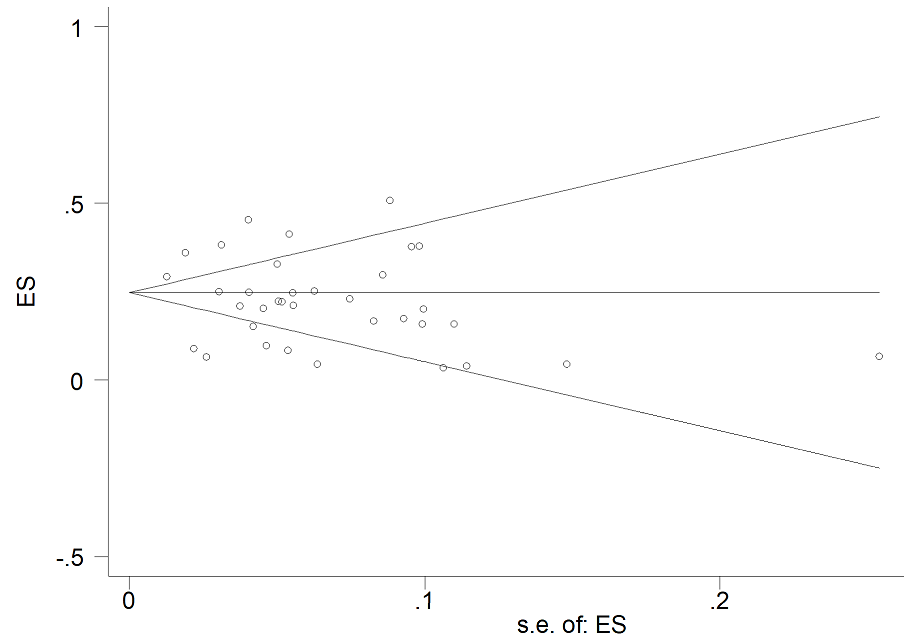


**Figure S3. Pooled prevalence of elevated troponins during hospital stay**


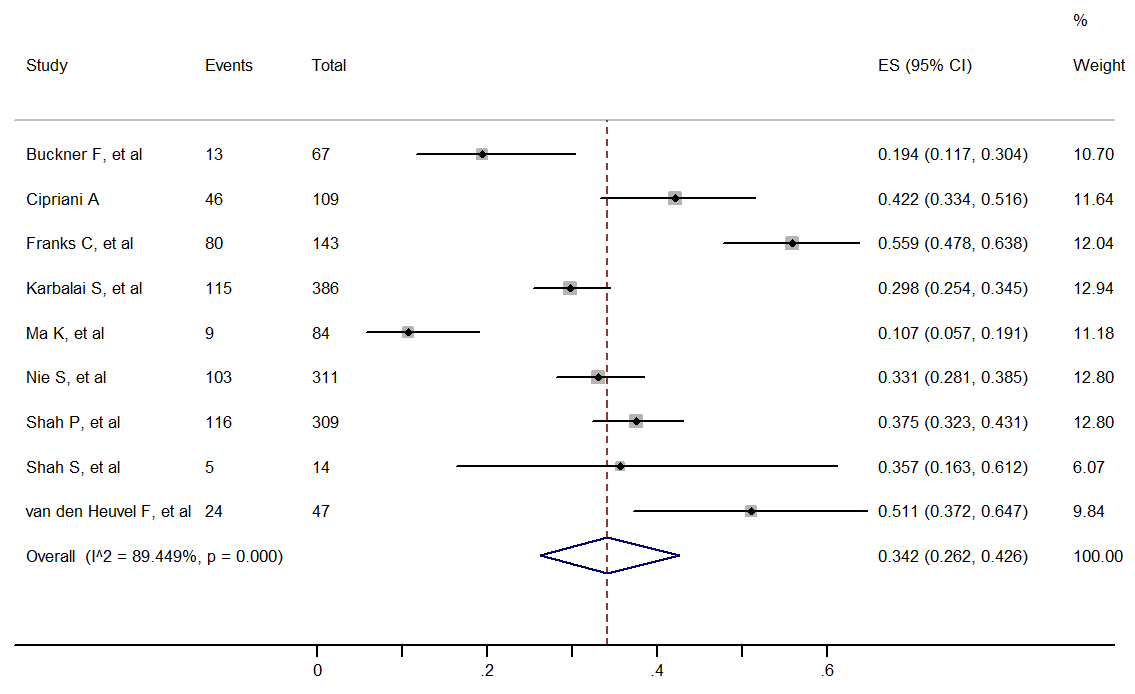


**Figure S4. Pooled prevalence of elevated troponins in patients admitted to intensive care unit**


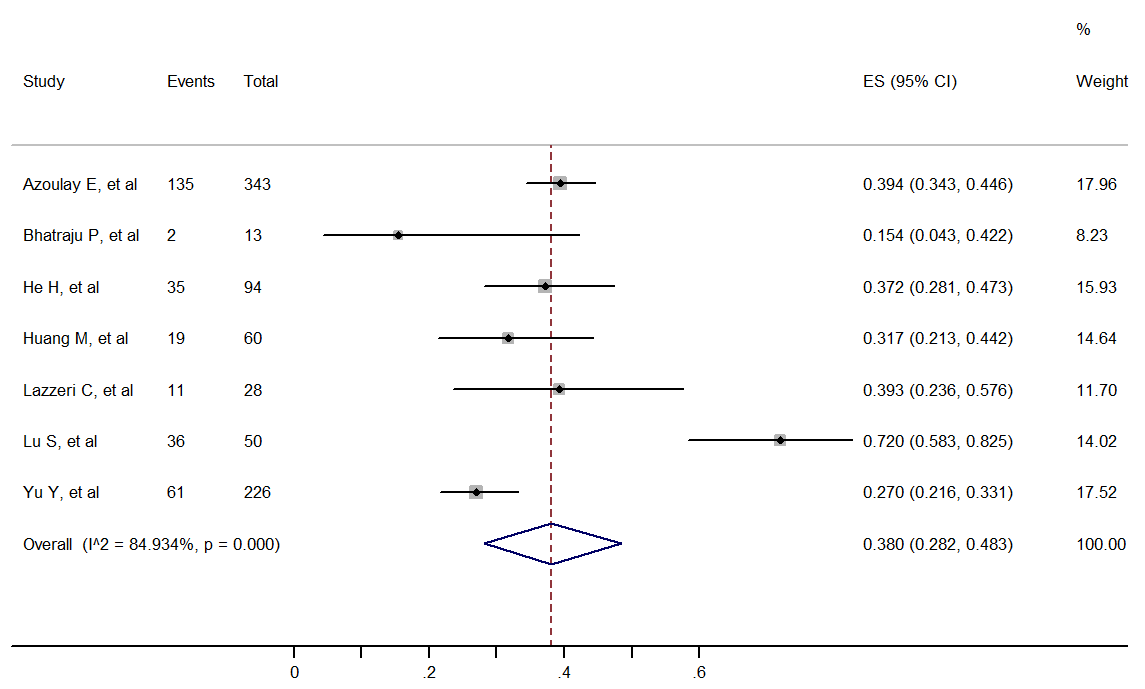


**Figure S5 (a) Funnel plot for assessing publication bias in the association between elevated admission troponins and mortality risk. (b) Funnel plot after applying the trim-and-fill method.**

1. **(b)**


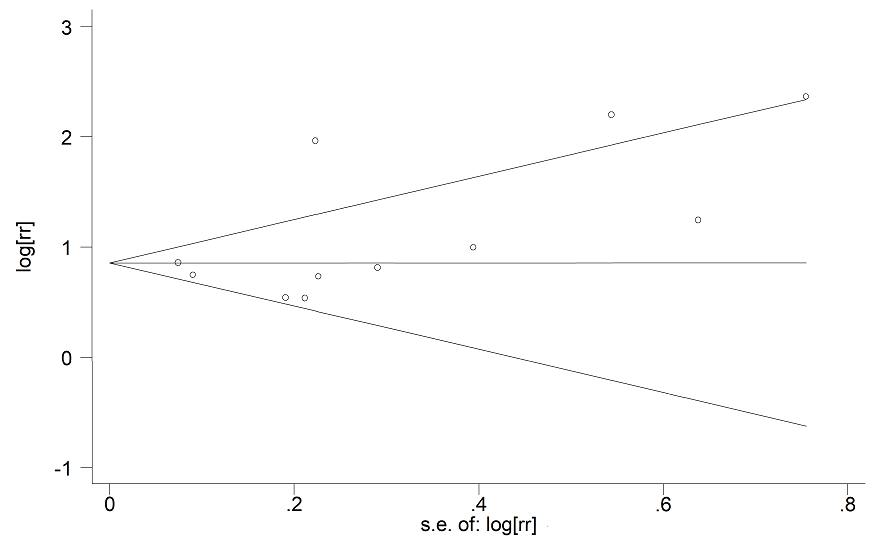

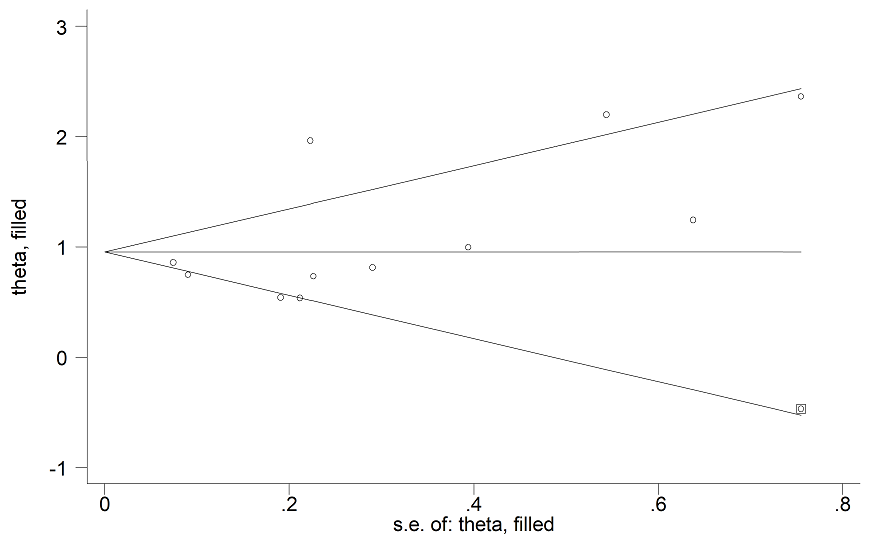

Supplement: Supplementary file 1 — Additional file 1: Table S1. Preferred Reporting Items for Systematic Reviews and Meta-Analyses (PRISMA) checklist. Table S2. Literature search strategy (PubMed as example). Table S3. Risk of bias assessment for studies on the prevalence of elevated troponin in patients hospitalised for covid-19. Table S4. Quality in Prognostic Studies (QUIPS) risk of bias assessment for studies on the association between elevated troponin and mortality. Table S5. Multivariable-adjusted association between elevated troponin and mortality in patients hospitalised for covid-19. Table S6. Subgroup analyses on the prognostic value of elevated troponins on admission for predicting death. Table S7. Covid-19 severity of illness classification. Figure S1. Pooled prevalence of elevated troponins on hospital admission. Figure S2. Funnel plot for assessing publication bias in the prevalence of elevated troponins on hospital admission. Figure S3. Pooled prevalence of elevated troponins during hospital stay. Figure S4. Pooled prevalence of elevated troponins in patients admitted to intensive care unit. Figure S5. (a) Funnel plot for assessing publication bias in the association between elevated admission troponins and mortality risk. (b) Funnel plot after applying the trim-and-fill method. [file 40560_2020_508_MOESM1_ESM.docx]
